
JOURNAL INFORMATION
==============================
NLM Title Abbreviation: Innov Aging
Journal ID: Innov Aging
NLM Journal ID: 101703706
Journal ID: innovateage

Innovation in Aging

EISSN: 2399-5300
Publisher: Oxford University Press

ARTICLE INFORMATION
==============================
PMCID: PMC9770034
DOI: 10.1093/geroni/igac059.697
Article ID: igac059.697
Article version: 1
Subjects: Abstracts, Session 3080 (Symposium), AcademicSubjects/SOC02600

NIA DIVISION OF BEHAVIORAL AND SOCIAL RESEARCH

Plude Dana National Institute on Aging, Bethesda, Maryland, United States

Dr. Plude will discuss research priorities for the Division of Behavioral and Social Research. Dr. Plude will also be available for small group discussion.

Publication date: 2022 Nov
Electronic publication date: 2022 Dec 20
Volume: 6
Issue: Suppl 1
Issue title: Program Abstracts from The GSA 2022 Annual Scientific Meeting, “Embracing Our Diversity. Enriching Our Discovery. Reimagining Aging.”
First page: 174
Last page: 174
Copyright: © The Author(s) 2022. Published by Oxford University Press on behalf of The Gerontological Society of America.
Copyright year: 2022
License: This is an Open Access article distributed under the terms of the Creative Commons Attribution License (https://creativecommons.org/licenses/by/4.0/), which permits unrestricted reuse, distribution, and reproduction in any medium, provided the original work is properly cited.
License URL: https://creativecommons.org/licenses/by/4.0/

Page count: 1

==============================

### NIA DIVISION OF BEHAVIORAL AND SOCIAL RESEARCH

Dana Plude, *National Institute on Aging, Bethesda, Maryland, United States*

Dr. Plude will discuss research priorities for the Division of Behavioral and Social Research. Dr. Plude will also be available for small group discussion.
